# Context matters: sexual signaling loss in digital organisms

**DOI:** 10.1002/ece3.1631

**Published:** 2015-08-18

**Authors:** Emily G Weigel, Nicholas D Testa, Alex Peer, Sara C Garnett

**Affiliations:** 1 Department of Integrative Biology, Michigan State University288 Farm Lane Road RM 203, East Lansing, Michigan, 48824; 2 BEACON Center for the Study of Evolution in Action, Michigan State UniversityEast Lansing, Michigan, 48824; 3 Department of Computer Sciences, University of Wisconsin-MadisonMadison, Wisconsin, 53706

**Keywords:** Digital evolution, genetic linkage, mate preference, population size, sexual signals

## Abstract

Sexual signals are important in attracting and choosing mates; however, these signals and their associated preferences are often costly and frequently lost. Despite the prevalence of signaling system loss in many taxa, the factors leading to signal loss remain poorly understood. Here, we test the hypothesis that complexity in signal loss scenarios is due to the context-dependent nature of the many factors affecting signal loss itself. Using the Avida digital life platform, we evolved 50 replicates of ∼250 lineages, each with a unique combination of parameters, including whether signaling is obligate or facultative; genetic linkage between signaling and receiving genes; population size; and strength of preference for signals. Each of these factors ostensibly plays a crucial role in signal loss, but was found to do so only under specific conditions. Under obligate signaling, genetic linkage, but not population size, influenced signal loss; under facultative signaling, genetic linkage does not have significant influence. Somewhat surprisingly, only a total loss of preference in the obligate signaling populations led to total signal loss, indicating that even a modest amount of preference is enough to maintain signaling systems. Strength of preference proved to be the strongest single force preventing signal loss, as it consistently overcame the potential effects of drift within our study. Our findings suggest that signaling loss is often dependent on not just preference for signals, population size, and genetic linkage, but also whether signals are required to initiate mating. These data provide an understanding of the factors (and their interactions) that may facilitate the maintenance of sexual signals.

## Introduction

Sexual signals are used to attract, assess, and secure mates. While sexual signaling is not ubiquitous, signals and associated preferences are extremely common across many taxa (Kirkpatrick and Ryan 1991; Andersson 1994; Getty 1998). Sexual signals can send information about potential mates, as well as benefits to a female and her offspring (Kotiaho and Puurtinen 2007; Lancaster et al. 2009). Despite these apparent benefits that suggest signaling systems would be preserved once they arise, signaling is frequently lost (Wiens 2001).

One reason that signals may be lost is that their production and evaluation can be costly (Endler 1983; Andersson 1994). Potential costs can either be direct, such as increased conspicuousness influencing predation, or more cryptic, such as loss of energy diverted to signaling or preference maintenance (Endler 1983; Andersson 1994). Furthermore, the magnitude of signaling costs can also vary depending on environmental factors, such as the availability of food (Hill et al. 2002), mates (Tinghitella et al. 2013), and abundance of predators or parasites (Zuk and Kolluru 1998; Johnson and Basolo 2003).

Although many of the costs to signaling have been considered in isolation, these factors can and will interact to shape the cost-benefit ratios of maintaining signals. What is unclear then are the specific contexts in which sexual signaling is lost in spite of its benefits. Several mechanisms have been proposed to explain how selection can overcome the costs of signaling, but ultimately the specific conditions determining signal maintenance or loss remain unclear (Wiens 2001).

Signaling systems appear to be surprisingly labile and are affected by a myriad of environmental, social, and genetic variables. Evidence suggests that physical and ecological changes in an organism's environment can alter signaling patterns and efficacy by changing the context in which signaling takes place (Schluter and Price 1993; Baird et al. 1997; Endler and Basolo 1998; Boughman 2002; Welch 2003; Maan et al. 2006; Reichard et al. 2009; Maan and Seehausen 2011). Likewise, changes to the social environment can impact signal loss. For example, recent work in *Teleogryllus* crickets demonstrates that social factors, such as male competition and female choice, are possible mechanisms of signal loss (Tinghitella et al. 2009). Finally, although less-thoroughly tested empirically, the genetic architecture of signaling organisms has the potential to play a large role in how easily signaling systems can deteriorate. Many sexual signaling models, such as Runaway (Lande 1981) and “Sexy Son” (Kirkpatrick 1985; Pomiankowski et al. 1991), as well as some Good Genes models (Schluter and Price 1993; Kirkpatrick 1996; Iwasa and Pomiankowski 1999), depend on a component of evolved genetic linkage between signal and preference traits, which may further rely on genetic components that regulate their expression, even for condition-dependent signals (Mead and Arnold 2004). Random factors such as genetic drift have therefore been suggested as possible causes for signal loss and species divergence, particularly in small populations (Lande 1981).

Although many variables have been suggested as causal in signal loss of natural organisms, the difficulty of generalizing across many organisms and contexts remains. To investigate the evolution and subsequent loss of sexual signaling under several conditions, we therefore utilized the digital life platform, Avida (Lenski et al. 1999; described in detail below).

Contrary to natural systems, Avida is a powerful tool that allows us to manipulate and study the interaction of several environmental, social, and genetic factors in real time. Avida also confers a unique advantage: Natural systems in which sexual signals are lost are studied after the proposed loss of traits (e.g., swordtails (Morris 1998), ducks *Anas* spp. (Omland 1996), pied flycatchers (Saetre et al. 1997), *Teleogryllus oceanicus* crickets (Tinghitella and Zuk 2009), and lizards *Phrynosomatidae* (Wiens 1999)). Here, we have the opportunity to study an evolved signaling population before, during, and after signal loss has occurred, independent of species-specific ecology and physiology that can mask general patterns. This level of detail, a rarity in biological systems, can elucidate the processes of signal loss in a simple and generalizable manner.

In order to study sexual signal loss and make generalizable inferences on more complex systems, we chose to focus on only the aspects that every signaling system must have, by definition:

1. Every population is necessarily made up of some number of individuals. Genetic drift alone suggests that population size has an effect on the maintenance of signaling.
2. Every population is necessarily made up of individuals with genotypes. Within these genotypes are genes that encode signals and preference. The nature and position of these genes within the genotype can therefore affect the likelihood of signaling system loss.
3. Every signaling population must necessarily be sending and receiving information. If such signals are proposed to have evolved because they conferred a mating advantage, differences in preference for signals should impact signal loss.
4. While sexual signaling may not be necessary for all organisms to reproduce, for many it is the only way to initiate reproduction. Whether signaling is required to contact potential mates or is simply optional may change the nature by which other factors interact.

Each of the above mechanisms has different implications for signal loss and may work in separate, but nonexclusive ways with one another. We therefore hypothesize that complexity in signal loss scenarios is due, at least in part, to the context-dependent nature of these mechanisms. Each mechanism has the potential to act differently given varying signaling requirements for reproduction; whether signaling is facultative or obligate can control how certain mechanisms affect signal loss.

Our Avida model allows us to test several hypotheses relating to the maintenance and loss of sexual signals in populations. First, small population sizes will be subject to greater effects of genetic drift, which should overcome sexual selection to maintain traits, regardless of whether signaling is required to attract mates. We predict that small population sizes will lead to higher amounts of signal loss in both obligate and facultative signaling populations. Second, we expect that the genetic architecture of organisms will affect the rate of signal loss. Genes colocated will experience less loss, particularly in populations under strong preference for the signal and those required to signal to find a mate. Third, we predict that a loss of preference leads to signal loss. With decreased or absent preferences, we expect to see signals lost, as sexual selection to maintain signals is weakened. Loss of signaling should then be more pronounced when signaling is not required for mating. Finally, we expect that signal loss will be much more modest in obligate systems, whereas facultative systems may experience more flexible levels of signal loss. The level of signal loss will still depend on the previous mechanisms, but will respond based on the context of whether signaling is obligate or facultative.

## Materials and Methods

### Avida

Here, we utilized the digital life platform, Avida (Lenski et al. 1999; described in detail in Ofria and Wilke 2004), to investigate the evolution and subsequent loss of sexual signaling under several conditions. Avida is a program in which populations of “digital organisms” (avidians) self-replicate, mutate, and compete for resources. Each avidian is given a set of instructions (a genome), which is passed on to its offspring. Variation in digital genomes arises through the processes of mutation and sexual recombination, allowing for remarkably complex genetic interactions, much like natural systems. Due to heritable genetic variation and selective forces present, Avidians are subject to evolution.

Avida is an established system in the study of many biological phenomena. The evolution of phenomena such as quorum sensing (Beckman et al. 2012), division of labor (Goldsby et al. 2014), ecological networks (Fortuna et al. 2013), multicellularity (Hessel and Goings 2013), prey intelligence (Wagner et al. 2014), and antipredator strategies (Fish et al. 2014) have all been studied in Avida. Our understanding of kin inclusivity (Johnson et al. 2014), host–parasite coevolution (Zaman et al. 2011), diversity in response to resource availability (Walker and Ofria 2012), recapitulation theory (Clune et al. 2012), temporal polyethism (Goldsby et al. 2012), and ecological and mutation-order speciation (Anderson and Harmon 2014) has been improved through studies in Avida. Specifically relevant to our study, Avida has lead to new insights into the study of the evolution of communication networks (Knoester and McKinley 2012), the evolution and maintenance of sexual reproduction (Misevic et al. 2010), the role of deleterious mutations in sexual populations (Covert et al. 2013), and hypotheses in runaway sexual selection and good genes (Chandler et al. 2013).

Avidians exist in a fixed-dimension, computational environment. Each organism occupied a single unique “cell” (in a toroidal gridspace) in a defined environment and competed for CPU cycles (energy used for replication) by completing tasks (mathematical functions). Which tasks were completed and how efficiently they were performed were defined by an organism's genome. Each genome was composed of a series of computer instructions (genes) that by default were sequentially executed and copied, instruction-by-instruction, to produce offspring. Each new organism was placed at a random location in the population's gridspace, killing the previous occupant of that cell. Each organism's genome was subject to random mutation at a rate of 3%, which may have increased or decreased the organism's ability to complete tasks, earn CPU cycles, and replicate. Additionally, space was limited; therefore, the faster a given organism earned CPU cycles and produced offspring, the more likely its genotype was to persist and spread in the population over time. Because these processes allowed for populations to experience selection and differential reproductive success between individuals over generations, individuals have fitness and populations evolve.

Using this digital and rapidly evolving system, we simultaneously observed and manipulated the strengths of several factors predicted to be important to the process and pattern of signal loss. In Avida, thousands of generations are generated in hours, and therefore, we could study the long-term, emergent behaviors of our system. For this study, we modified Avida to include two new binary instruction genes: a “mate-signal” gene that represents possessing and sending a signal, and “mate-receive”, which was modifiable by our preference parameter and used to detect signals and mate accordingly.

### Organism genomes

We chose to utilize sexually reproducing organisms within Avida. In addition to typical asexual avidian mutation parameters (such as insertions, deletions, and copy mutations), the novel use of sexual organisms allowed for potential genome modification by sexual recombination. Initial populations began as clones of a single genome containing one signaling gene and one receiving gene; these two genes were either adjacent (linked) or at the furthest possible distance apart in the circular genome (unlinked) in treatments to test for the effects of genetic linkage on signal loss. Each genome was of a fixed size, including genes for signaling and receiving, replication, and genes which do not code any information. Because only signaling, receiving, and replication behaviors were explicitly coded by genes, all other behaviors exhibited by each organism's descendants must be evolved.

At the beginning of each run, every cell in the environment was injected with a clone of the default organism, that is, an organism which has both the signaling and receiving instruction genes (“mate-signal” and “mate-receive,” respectively). Multiple copies of a gene (created through copy mutations or through recombination in previous generations) can allow for the possibility of increased expression of genes within a single organism's genome; however, the duplication of signaling genes would require the replacement of other genes in the genome, due to the genome's fixed size.

### Signaling

When *mate-signal* was expressed, a message was broadcasted to nearby organisms. Receivers were capable of “remembering” only the last eight signals received, forgetting the oldest signal when a new signal is added. Each signal sent contained an ID tied to the cell location of the signaler (“mateID”) to be used in the mating phase to locate chosen mates. When an organism expressed *mate-receive***,** it chose a mate from among the organisms whose signals it has received, modified by strength of preference (detailed below). This decision was made based on “merit,” a real-time proxy for fitness akin to condition-dependent signaling; the higher the merit, the better the organism is at utilizing resources from its environment. The highest-merit signaler was chosen to mate, and its mateID was remembered by the receiver to later select the correct mate within the mating arena. Because a number of signals could have been sent by various males, and females could receive signals from many males and needed to choose from among them, this mimicked a lek in natural populations. Once a mate was chosen, all currently remembered signals were forgotten. In order to differentiate between organisms that cannot receive signals and those simply not in the presence of signaling organisms, the receiver returns a special value indicating that no message was received. The execution of both signaling and receiving genes was costly in terms of both CPU cycles and time required to execute each command.

### The birth chamber

The execution of the sexual recombination mating instruction *divide-sex* caused the organism to enter a mating arena known in Avida as the “birth chamber.” Here, parent organisms first divided like the default asexual avidian to create a “gamete,” which was stored in an array indexed by its parent's cell ID. These gametes were stored until overwritten by another entry for that cell ID, that is when a new organism is born into that cell location within the environment, displacing the original organism.

If the receiving organism had successfully received a signal (i.e., it has been exposed to mate-signal, and then executed mate-receive), it selected the stored gamete matching the cell ID of the chosen mate, and both gametes sexually recombined to produce one offspring. If the organism had *not* received a signal (or lacks the gene to receive signals), the effects depended on whether signaling is required for mating: When signaling was obligatory for mating, mating fails. However, if signaling was *not* obligatory (i.e., facultative) for mating, a cell location is chosen at random from the population, and the gamete stored for that location is used for recombination. The specific conditions therefore represented populations where signaling was required (or not) to find mates; thus, when signaling is facultative and signals are not received, random mating resulted. Once mating occurred, each newly born offspring was placed in a random cell location in the environment. If no empty cell was available in the population, replication results in the replacement of another organism in that cell location within the population.

It is also worth noting that when a gamete was chosen from storage, there are two rare cases that might have resulted in the gamete not matching the organism currently in the corresponding cell. First, if no gamete was stored for a cell location, mating failed (one gamete cannot produce a viable offspring). Second, a lag between a signal and the signaler's own division may have caused the gamete of a cell's deceased former occupant, or a cell's new occupant, to be used. The first case could only occur before any organism has divided in that cell (and so was restricted to early in a run); the second case of using a deceased organism's gamete is possible in a diverse array of sperm storing females in the animal kingdom, including many birds (Liem et al. 2001), insects (Klowden 2003), pigs (Suarez 2002), whale sharks (Schmidt et al. 2010), and snapping turtles (Galbraith et al. 1993). Thus, these cases, although biologically relevant, were fairly rare.

### Initial states and experimental treatments

The historical state of all populations was evolved under obligate signaling, and the obligation to signal was either retained or removed when the experiment began. This represented a known historical branching point in a phylogeny with two descendant species: one which must signal to mate, and one for which signaling was not required.

In order to standardize and control for initial population differences across all treatments, the identical evolved population of organisms from the historical population was used to start each combination of treatments. Each replicate population then evolved under a linkage, population size, and a strength of preference treatment along with either obligatory or facultative signaling to mate. Linkage treatments were binary (either linked or unlinked signaling-receiving genes), and the maximum population size in each environment was manipulated at 200, 100, 50, 25, or 10% of the default population size in Avida, or approximately 7140, 3600, 1806, 900, and 342 individuals per population. Additionally, strength of preference was manipulated at 100, 75, 50, 25, or 0%. Strength of preference represented the likelihood of guaranteed mating success for the signaler with the highest merit for which a signal was received, where 100% represented an extremely strong preference for the highest merit, and 0% represented no preference for any signal received, that is, random mating with any individual in the population, regardless if signaling or not. Note that under the obligate signaling condition, because signaling was required for mating, a lack of signaling meant no mating could occur, and the population therefore went extinct.

Each replicate (per treatment, *n* = 50) lasted 50,000 updates (approximately 700 generations). We implemented the default configurations for Avida: 100% probability of sexual recombination (random, modular swapping of instructions) per mating, 5% probability of insertion–deletion mutations, and 0.75% probability of copy mutations. There were two linkages, five population sizes, and five strength of preference treatments, each with 50 replicate populations, used for both populations required and not required to signal. This yielded 150 unique combinations (7500 Avida runs) generated using computing resources at Michigan State University's High Performance Computing Center.

### Statistical analyses

All statistical analyses were conducted using R statistical software (R Core Team, 2015, version 3.3.1). Here, signaling trait loss is defined as the inability for organisms in a population to signal. Signaling loss is therefore a binary, rather than continuously variable, trait in our populations: an organism either is or is not able to signal (i.e., both an organism who duplicates the signal gene and one who is still able to signal after gene degradation would be considered to have maintained signal). This binary signaling distinction is analogous to natural systems, such as those of *Teleogryllus oceanicus* (Tinghitella and Zuk 2009), where males can either signal or are mute. Signal loss was calculated by combining the total percentage of individuals in a population that could only receive the signal and those that could neither signal nor receive; remaining individuals (signalers and signalers/receivers) therefore constituted signalers left. As signal loss was measured for the same population over time, we assessed the effect of signal preference, genetic linkage, population size, and signaling regime on signal loss. This model, a repeated-measures analysis of variance (ANOVA), allowed us to compare the effect of each variable, including their interactions among one another over time, while still accounting for lack of independence within lines. Repeated-measures ANOVA were run under two conditions: with variables treated as continuous and as categorical. While our experiment was designed with categorical treatment groups, the significance of the results and relative importance of the predictor variables did not change when analyzed as continuous or categorical variables. We therefore report the most conservative significance values from continuous variable analysis. Finally, Levene's test for equality of variances was utilized to analyze the effect of population size on variance in signal loss.

## Results

According to our hypotheses, there are four potential mechanisms by which our organisms could lose signaling phenotypes. Data were recorded for the historical signaling regime (obligate) and continued to be collected in the two branched, descendant populations where signaling was now either obligate or facultative. Listed in order of their *F*-value, the mechanisms of signal loss are as follows: preference for signal, obligation to signal, genetic linkage, and population size. We found that all first- and second-order interactions have a significant effect on signal loss (Table1).

**Table 1:** Repeated-measures ANOVA results (all data)

	*F*-value	*P* value
Effect		
Population size	78	<2E-016***
Genetic linkage	6112	<2E-016***
Strength of preference for signal	311,300	<2E-016***
Obligation to signal	32,630	<2E-016***
Interactions		
Population size	5	0.027783*
Linkage	–	–
Population size	21	4.01E-006***
Preference	–	–
Linkage	706	<2E-016***
Preference	–	–
Population size	155	<2E-016***
Obligation	–	–
Linkage	2368	<2E-016***
Obligation	–	–
Preference	10,480	<2E-016***
Obligation	–	–

Significance (P) values are as follows: * = 0.01, *** = <0.001.

**Figure 1:**
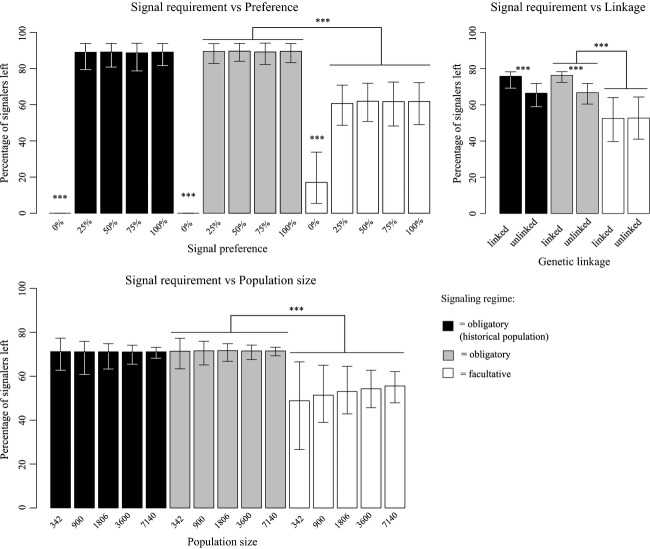
Percentage of signalers remaining is based on preference for signal (top left), population size (bottom), and genetic linkage (top right), further subdivided by signaling regime. Note that both a lack of preference and facultative signaling (top left) significantly increase signal loss. Genetic linkage (top right) tends to preserve signaling systems, specifically for obligate signalers, although facultative signaling eliminates this trend. Finally, variance in sexual signal loss increases as population size decreases (bottom left) and is more pronounced when signaling is facultative. No population size tested has been shown sufficient to significantly reduce signal loss. Bars show 95% confidence intervals (repeated-measures ANOVA, ***P < 0.001; **P < 0.01; *P < 0.05). Significance (P) values are as follows: * = 0.01, *** = <0.001.

Contrary to our expectations, only the lack of preference for signals led to their complete loss. We found that the level of signal preference greatly affected signal loss (*F* = 311,300, *P* < 0.0001). All second-order interactions with preference are strongly significant, but the interaction of preference and obligation to signal is the strongest by at least two orders of magnitude (Fig.1).

The effect of genetic linkage on signal loss was also shown to be strong (*F* = 6112, *P* < 0.0001), although this effect is magnified when linkage covaries with the obligation to signal (*F* = 2368, *P* < 0.0001; Fig.1).

**Figure 2:**
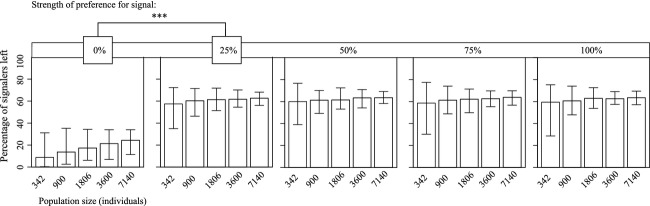
The effect of preference for signal on signal loss in several population sizes. As expected, greater variance is seen in smaller population sizes where the effect of genetic drift is more pronounced. Note that only a lack of preference leads to substantial levels signal loss. Bars show 95% confidence intervals (repeated-measures ANOVA, ***P < 0.001; **P < 0.01; *P < 0.05).

**Figure 3:**
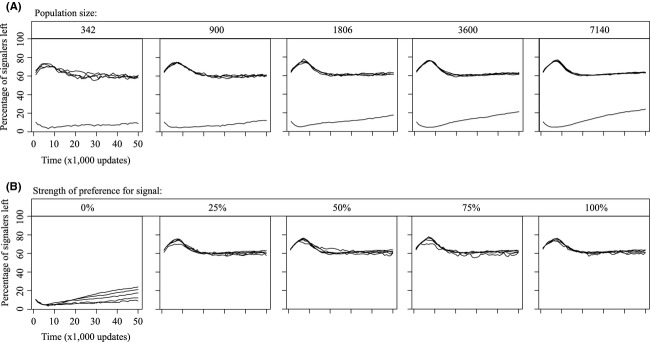
Loss of signaling over time for facultative signaling populations that vary in size (A) and preference for signal (B). Lines represent the mean value for signal loss over time in various treatment groups. As expected, lower population sizes (A) increase variation and cause signals to be lost more unpredictably. Signal preference (B) appears to consistently maintain signaling when present, but a lack of preference leads to signal loss.

Population size was shown to increase the variability of signal loss with decreasing populations (Fig.2). As population sizes decreased, signal loss responded in two related, but important, ways: We observed a greater overall signal loss (*F* = 78.29, *P* < 0.0001), but Levene's test for equal variances also revealed a significant increase in the mean variance of signal loss (*F* = 237.87, *P* < 0.0001). Variation in signal loss due to small population sizes is visible as increased scatter in mean signal loss through time (Fig.3). All second-order interactions of population size with other mechanisms are significant, although their effect sizes are quite small (Table1). As seen in Fig.2, population size has relatively little effect on signal loss until preference for signal is decreased to zero. Population size only affects signal loss in the absence of preference.

A lack of preference (i.e., preference = 0) behaves very differently than when preference is present. Therefore, we conducted further analyses that included only preferences greater than 0. Many previously strong effects lose significance in this second model (Table2). For instance in the “nonzero preference” analysis, preference strength (*F* = 1.704, *P* = 0.1918) and its higher-order interactions with population size (*F* = 0.47, *P* = 0.4931) and genetic linkage (*F* = 0.419, *P* = 0.5175) no longer negatively affect signal loss. The obligation to signal (and all of its interactions) becomes the strongest modifier of signal loss in this analysis, followed by genetic linkage and population size (Table2).

**Table 2:** Repeated-measures ANOVA results (nonzero strength of preference only)

	*F*-value	*P* value
Effect		
Population size	151	<2E-016***
Genetic linkage	46,960	<2E-016***
Strength of preference for signal	2	0.1918
Obligation to signal	324,100	<2E-016***
Interactions		
Population size	27	1.82E-007***
Linkage	–	–
Population size	0	0.4931
Preference	–	–
Linkage	0	0.5175
Preference	–	–
Population size	380	<2E-016***
Obligation	–	–
Linkage	19,270	<2E-016***
Obligation	–	–
Preference	35	3.68E-009***
Obligation	–	–

Significance (P) values are as follows: *** = <0.001.

Only very few combinations ever appeared to completely, or even partially, eliminate signaling. Furthermore, persistence of signaling appeared to be relatively stable through time; it was either lost almost immediately or not at all (Fig.3). In all signaling regimes, a lack of preference had the potential to eliminate signaling. Lack of preference meant that organisms no longer prefer to accept any particular signal on the basis of merit; thus, signals, and the “quality” of the signaler that they convey, were ignored during mating. For the obligate signaling regime, lacking a preference for the signal provided an interesting case where receivers should have mated randomly with respect to signal quality, but because of the obligation to signal, receiving organisms could not initially identify mates. Because no mates could be identified, no mating occurred, and the population rapidly went extinct. For facultative signalers, a lack of preference was not deadly, but rather caused the signaling phenotype to be selectively neutral. Facultative signalers with no preference were especially prone to signal loss in low populations, where genetic drift was strongest (Fig.3).

## Discussion

Our work provides an important first step in concurrently testing the relative strengths of several factors proposed to lead to sexual signal loss. Here, we show that whether or not an organism needs to signal to initiate mating has a context-dependent relationship with genetic linkage, population size, and the strength of preference in determining likelihood of signal loss. We introduce a new variable, the obligation to signal, as a formerly underappreciated, but potentially important, factor in whether signals will be lost. Genetic linkage, population size, the strength of preference, and whether signaling is obligatory all affect rates of signal loss in populations, but are contextually variable and interactive. The context-dependent nature in which these factors interact shows how difficult it may be to perturb populations into signal loss, as no single factor was sufficient to drive signaling in all populations to complete loss. Furthermore, the addition of whether signaling is required to find a mate is important as it changes the magnitude of each of these factors in several different combinations.

Consistent with natural systems, strength of preference plays a large role in maintaining signals. Relatively weak preferences were enough to maintain sexual signals; however, populations in which preference was absent (i.e., strength of preference was zero) experienced substantial, and in some cases, total signal loss (Fig.2). Such a strong response suggests that preference plays an important role in the maintenance of sexual signals (Figs1 and 2). This suggests that the strength of sexual selection, even when relatively weak, is enough to override other mechanisms of evolution. Examples from the literature suggest that variation in female preference facilitates signal loss (Tinghitella and Zuk 2009), which may explain the abundance of cases where male traits are lacking and female preference is weakened (Endler and Houde 1995; Omland 1996; Morris 1998). Having a complete evolutionary history for signaling populations has allowed us to demonstrate definitively that loss of female preference can drive the loss of signaling.

As with *Heliconius* butterflies (Merrill et al. 2011), both the genetic architecture of signaling traits and the behaviors they encode are important for the evolution and maintenance of sexual signals. Models and empirical evidence show that mating with males displaying preferred signals provides benefits; thus, the co-occurrence of signal and preference genes should increase over evolutionary time (Jones et al. 1998; Welch et al. 1998; Whitlock et al. 1998; Head et al. 2005). For signals to be lost, the reverse could be true: Breaking genetic associations between signaling traits, not just the loss of genes themselves, could facilitate signaling lost. Additionally, whether populations were required to signal modulated the effects of linkage. Signal loss was approximately equal between linked and unlinked traits under facultative signaling. Under obligate signaling, however, a lack of genetic linkage promoted signal loss. Our results show incorporating the requirement to signal is certainly important, but has been previously overlooked when considering the impact of genetic linkage on signal loss.

The impact of population size, particularly in the face of genetic drift, has been implicated as an important driver of a diverse array of population-level effects. While drift still occurs within large populations, the effects are certainly exaggerated in smaller populations. We show that as population size decreases, variability in – and likelihood of – signal loss across populations increases (Fig.2). This pattern held true whether or not signaling was required to find mates. The degree of signal loss in facultative signaling populations was always equal or greater than that of obligate signaling populations across all treatments. Thus, we have direct evidence suggesting that genetic drift is an important force driving signal loss with fewer individuals. Our findings agree with long-standing work citing bottlenecks and small population sizes for reduced genetic variability in populations (Nei et al. 1975; Lande 1976). Future work examining a wider range of population sizes, particularly those much smaller, will strengthen this relationship.

Finally, this study highlights the important distinction that many organisms do not necessarily need to signal to find mates, but only to *assess* mates. In some cases, a loud call or conspicuous odor is necessary to attract mates over long distances, while additional elements of signals (or even new signals) may be used over shorter ranges to communicate supplementary information (Borgia 1995; Ringo 1996; Johansson and Jones 2007; Tinghitella and Zuk 2009). The clear implications of the requirement to signal necessitates that it be accounted for while studying signal loss, particularly in multimodal or long-range signaling systems.

This experiment did not address the potential mechanisms by which extremely small population sizes may affect signal loss. Mate search, for example, is an important consideration, particularly at small population sizes and among closely related species. Our Avidians do not experience a spatial, sequential search for mates which might be impacted to a greater extent by mate search costs and Allee effects at small population sizes (McCarthy 1997). Nonetheless, conservation research has shown that the loss of genes and decreased genetic diversity at small population sizes matches theoretical predictions (Montgomery et al. 2000). Our findings therefore strengthen the implications of future Avida studies to empirically investigate the interactive effects of drift (at small population sizes) and preference in speciation as proposed by Uyeda et al. (2009). Given considerations of both potential signal loss and speciation dependent on population size, it is important that future work on sexual signal loss addresses both the strength of drift and female preference, particularly when addressing conservation questions.

In addition to drift and female preference, the obligation to signal and degree of genetic linkage are important considerations in determining the likelihood and consequences of signal loss in natural populations. As addressed in Mendelson and Shaw (2012), many closely related species use signals during courtship to determine species identity, which may be dependent on, or independent of, the cues which communicate mate quality. Thus, obligations to signal in some populations may serve an important role in mate recognition to reduce gene flow between populations. However, facilitating the maintenance of distinct species may not always be ecologically adaptive for survival (Grant and Grant 1992; Semlitsch and Reyer 1992; Veen et al. 2001; Pfennig 2007); thus, signal loss in many cases may not be a detriment, but an advantage, under certain environmental conditions.

Furthermore, the degree of genetic linkage between preference and signaling trait is key in the *evolution* of signals via runaway sexual selection (as reviewed in McNiven and Moehring 2013). Given that organisms may be along the way to developing close genetic linkage in traits, and that these signals may divide species, how closely genes are linked may modulate the likelihood of signal *loss*, and its consequences, including hybridization.

We intentionally excluded some ecological factors in our comparisons of signaling populations. By only accounting for the factors that by definition exist in every signaling system, we are left with a model that can be used to make inferences on other systems, independent of species-specific ecological conditions. Further studies could therefore assess more specific ecological factors and how they interact with the basal, generalizable model. For example, predation can be an important selective force that shapes whether and how organisms signal, as well as the costs and benefits of search and assessment of mates. Implementing predation costs to signalers and receivers in Avida may yield alternate patterns of signal loss, as sexual and natural selection may then act in opposition. Additionally, although signals in our study are costly, the levels of intrinsic cost of the signal are static, but may show different patterns of loss if costs increased or decreased. The addition of any of these factors may change signaling regimes and be more applicable to certain biological models.

Overall, our data suggest that signal loss in natural populations should be rare. Paradoxically, we are now finding sexual signal loss to be more common in nature than once thought. It is unknown whether this is a function of increasing frequency of signal loss across taxa, or simply the ability and awareness to consider the losses of signals along with their gains. Should signal loss indeed be an increasing biological phenomenon, responses to environmental change are likely drivers. Social environments, as well as the ability to send and receive signals, may be more variable now due to rapidly changing environments (Candolin and Wong 2012), which may facilitate population-level signal loss. Within-population variation within the variables considered in our study, as well as behavioral plasticity in response to environmental change (such as in female choosiness; Tinghitella et al. 2013), could facilitate signal loss. Future work will need to consider how within-population variation in response to both the social and physical environment effects signal maintenance and loss.

Predicting signaling behavior responses to anthropogenic change may necessitate incorporation of ecological context. Human-induced disturbances can have a profound impact on the ecology of a variety of organisms, and mating in particular may be sensitive to ecological changes that render a given signaling modality nonadaptive. If rapid ecological change makes it more difficult to transmit or receive signals, organisms may be unable to identify, locate, or assess mates. Factors such as noise or light pollution have already been implicated in reducing the effectiveness of signals, which can result in changes in signaling (Cartwright et al. 2014; Costello and Symes 2014; Kunc et al. 2014; Radford et al. 2014). Knowing how factors influencing sexual signaling loss interact can help us predict population responses to environmental change, and whether responses will result in the loss of signals alone, or the species dependent on signals for mating.
